# Expanding of Life Strategies in Placozoa: Insights From Long-Term Culturing of *Trichoplax* and *Hoilungia*

**DOI:** 10.3389/fcell.2022.823283

**Published:** 2022-02-09

**Authors:** Daria Y. Romanova, Mikhail A. Nikitin, Sergey V. Shchenkov, Leonid L. Moroz

**Affiliations:** ^1^ Institute of Higher Nervous Activity and Neurophysiology of RAS, Moscow, Russia; ^2^ Belozersky Institute for Physico-Chemical Biology, Lomonosov Moscow State University, Moscow, Russia; ^3^ Kharkevich Institute for Information Transmission Problems, Russian Academy of Sciences, Moscow, Russia; ^4^ Department of Invertebrate Zoology, Faculty of Biology, Saint Petersburg State University, Saint Petersburg, Russia; ^5^ Departments of Neuroscience and McKnight Brain Institute, University of Florida, Gainesville, FL, United States; ^6^ Whitney Laboratory for Marine Biosciences, University of Florida, St. Augustine, FL, United States

**Keywords:** Placozoa, fiber cells, immunity, aging, development, nervous system evolution, regeneration, behavior

## Abstract

Placozoans are essential reference species for understanding the origins and evolution of animal organization. However, little is known about their life strategies in natural habitats. Here, by maintaining long-term culturing for four species of *Trichoplax* and *Hoilungia*, we extend our knowledge about feeding and reproductive adaptations relevant to the diversity of life forms and immune mechanisms. Three modes of population dynamics depended upon feeding sources, including induction of social behaviors, morphogenesis, and reproductive strategies. In addition to fission, representatives of all species produced “swarmers” (a separate vegetative reproduction stage), which could also be formed from the lower epithelium with greater cell-type diversity. We monitored the formation of specialized spheroid structures from the upper cell layer in aging culture. These “spheres” could be transformed into juvenile animals under favorable conditions. We hypothesize that spheroid structures represent a component of the innate immune defense response with the involvement of fiber cells. Finally, we showed that regeneration could be a part of the adaptive reproductive strategies in placozoans and a unique experimental model for regenerative biology.

## Introduction

Placozoans are essential reference species to understand the origins and evolution of the animal organization. Despite the long history of investigations, Placozoa is still one of the most enigmatic animal phyla. Placozoans have the simplest, among-free living animals, body plan—three cell “layer”s organization (Schulze, 1883; Metschnikoff, 1886; Noll, 1890; Graff, 1891; Metschnikoff, 1892; Stiasny, 1903; Ivanov, 1973; Rassat and Ruthmann, 1979; Dogel, 1981; Malakhov and Nezlin, 1983; Okshtein, 1987; Malakhov, 1990; Smith et al., 2014; Mayorova et al., 2019; Romanova, 2019; Smith et al., 2019; Romanova et al., 2021; Ruthmann et al., 1986; Ivanov et al. 1982), but surprisingly complex behaviors (Kuhl and Kuhl, 1963; Kuhl and Kuhl, 1966; Seravin and Karpenko, 1987; Seravin and Gudkov, 2005a; Eitel and Schierwater, 2010; Eitel et al., 2011; Smith et al., 2015; Senatore et al., 2017; Armon et al., 2018; Zuccolotto-Arellano and Cuervo-González, 2020; Romanova et al., 2020b) with social feeding patterns (Okshtein, 1987; Fortunato and Aktipis, 2019).

The phylum Placozoa contains many cryptic species because differences in morphological phenotypes are minor. The broad sampling across the globe revealed ∼30 haplotypes (Aleshin et al., 2004; Eitel and Schierwater, 2010; Miyazawa et al., 2012; Eitel et al., 2013; Schierwater and DeSalle, 2018; Miyazawa et al., 2021), based upon the mitochondrial 16S. In an initial molecular genetic diversity survey, Voigt et al. (2004) assigned the original Grell strain as the mitochondrial 16S haplotype H1, equal to the classical *Trichoplax adhaerens* (Schulze, 1883).

Among other haplotypes described so far, the H13 haplotype has been recognized as a separate species and genus—*Hoilungia hongkongensis* (Eitel et al., 2018). *Polyplacotoma mediterranea* is the third formally described genus of Placozoa (Osigus et al., 2019). Moreover, emerging data related to genomics, physiology, feeding, and ecology suggest that the H4 haplotype is a separate species of *Hoilungia* (=*Hoilungia* sp.). Similarly, the H2 haplotype can also be viewed as a distinct species of *Trichoplax* (=*Trichoplax* sp. - (Laumer et al., 2018; Schierwater and DeSalle 2018). Therefore, we refer to these four cultured haplotypes in the current manuscript as different species.

Placozoans can predominantly be collected from tropical and subtropical regions (Ueda et al., 1999; Signorovitch et al., 2006; Pearse and Voigt, 2007; Eitel and Schierwater, 2010; Nakano, 2014; Maruyama, 2004; Eitel et al., 2011); they live in a wide range of salinity (20–55 ppm), temperature (11–27°C), depth (0–20 m), and pH (Schierwater, 2005; Schierwater et al., 2010; Eitel et al., 2013). However, their lifestyles are essentially unknown. The morphotypes and reproductive strategies of placozoans vary depending on feeding conditions. Pearse and Voigt, (2007) had suggested that placozoans may be opportunistic grazers, scavenging on organic detritus, algae, and bacteria biofilms.

Long-term culturing helps to explore the life histories of placozoans further. Most of the knowledge about placozoans had been obtained from culturing of just one species, *Trichoplax adhaerens* (Pearse, 1989; Signorovitch et al., 2006; Eitel and Schierwater, 2010; Eitel et al., 2013; Heyland et al., 2014). Both rice and algae had been used as alternative food sources. For example, the feeding substrates could be: *Cryptomonas* (Grell, 1972; Ruthman, 1977), red algae *Pyrenomonas helgolandii* (Signorovitch et al., 2006), green algae (*Ulva sp*; Seravin and Gerasimova, 1998), or a mix of green, *Nannochloropsis salina*, and red algae, *Rhodamonas salina*, *Pyrenomonas helgolandii* (Jackson and Buss, 2009; Smith et al., 2014), as well as yeast extracts (Ueda et al., 1999).

In addition to the disk-like flattened placozoan bodyplan, various culturing conditions resulted in different morphological forms. Thiemann and Ruthmann (1988); Thiemann and Ruthmann (1990); Thiemann and Ruthmann (1991) described several spherical structures and swarmers as asexual/vegetative reproductive stages. These original observations have been made on *T. adhaerens* only. There are no reports about similar structures and functions in other species/haplotypes of placozoans. Here, by maintaining long-term culturing for four species of *Trichoplax* and *Hoilungia*, we provided additional details about feeding and reproductive adaptations relevant to placozoan ecology and immune mechanisms.

## Material and Methods

### Culturing of Placozoans

We used axenic clonal cultures of four species of Placozoa: *Trichoplax adhaerens* (Grell’s strain Н1, from the Red Sea), *Trichoplax* sp. (H2 haplotype, collected in the vicinity of Bali island), *Hoilungia* sp. (H4 haplotype, collected in coastal waters of Indonesia), and *Hoilungia hongkongensis* (H13 haplotype, found in coastal waters of Hong Kong). We maintained all species in culture for 3–5 years (2017–2021), allowing long-term observations and adjustments of culture conditions for each haplotype/species.

We cultured H1, H2, and H13 in closed Petri dishes with artificial seawater (ASW, 35 ppm, pH 7.6–8.2), which was changed (70% of the total volume) every 7–10 days. On average, 5–10 Petri dishes were used every week with 200–300 animals on each plate. Monitoring and observation occurred daily.

**FIGURE 1:**
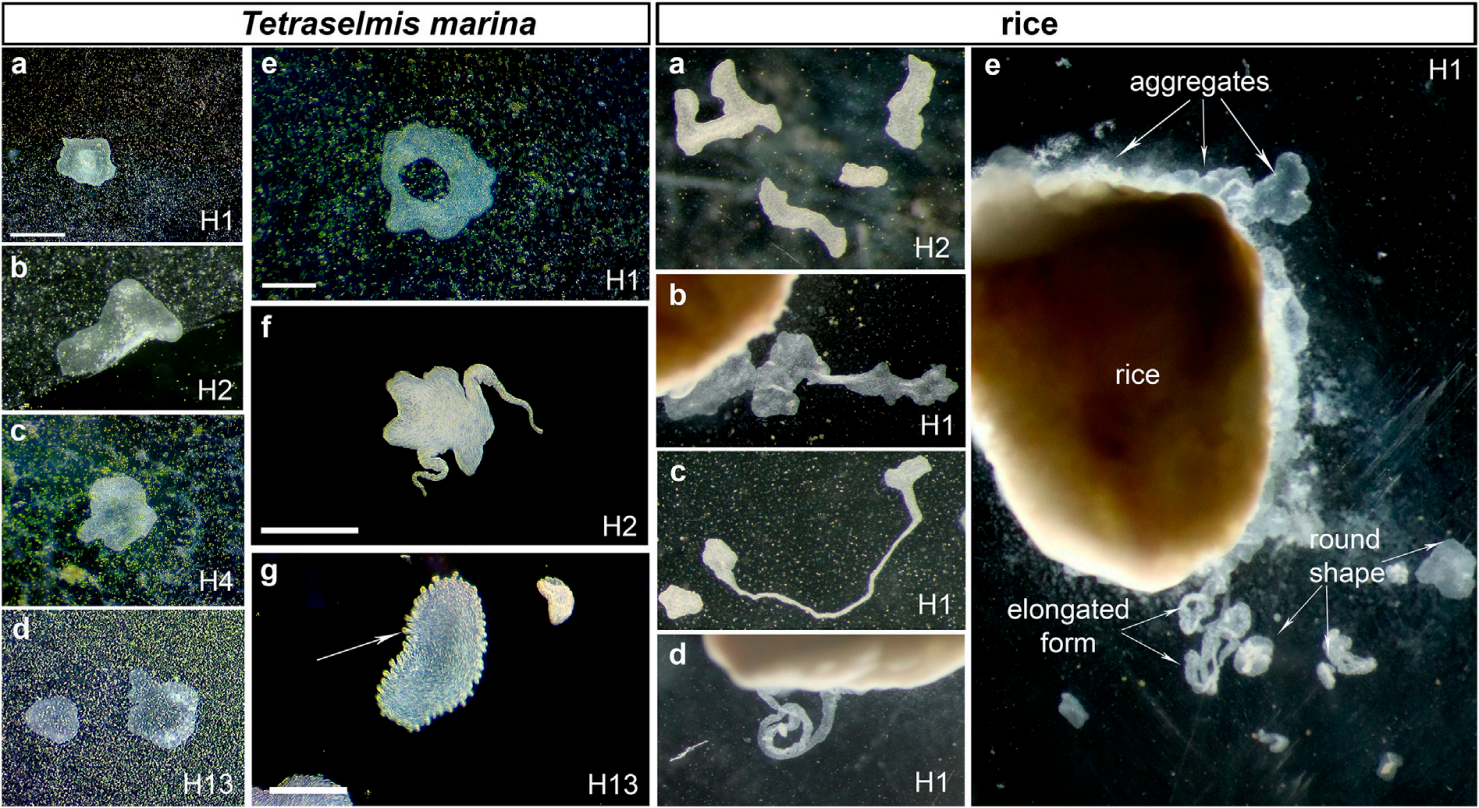
The diversity of placozoan body forms. Illustrated examples from long-term culturing of different haplotypes (indicated in each photo) on two feeding substrates (*Tetraselmis marina* and Rice boxes). *Tetraselmis marina* box shows both canonical placozoans body shapes **(A–D)** and unusual morphologies (animals with a “hole” in the middle of the body (**E** and Supplementary Video S1), elongated “pseudopodia”-like structures (Supplementary Figure S3), and animals with numerous small ovoid formations in the rim area **(G)**. The ‘rice box’ illustrate examples of different body morphology in cultured placozoans using different feeding substrate. Rice grains support a stable population growth rate but often facilitate the formation of aggregations of animals around grains (rice box **A–D**: different shapes of placozoans **(A–B)**, including those with highly elongated forms **C,D**). Scale bar: *Tetraselmis marina* box: 500 μm; Rice box: grain: 1 mm.

A suspension of the green alga *Tetraselmis marina* (WoRMS Aphia, ID 376158) was added to the culture dishes. When the biofilm of microalgae became thinner or depleted, freshly prepared, 1–2 ml suspension of *T. marina* could be added to the culture dishes weekly. Mixtures of other algal clonal strains were also occasionally used (for example, the cyanobacteria *Leptolyngbya ectocarpi* (WoRMS Aphia, ID 615645) and *Spirulina versicolor* (WoRMS Aphia, ID 495757), the red algae such *Nannochloropsis salina* (WoRMS Aphia, ID 376044) or *Rhodomonas salina* (WoRMS Aphia, ID 106316). H1, H2, and H13 were maintained at the constant temperature of 24°С and natural light in environmental chambers (see Modes 1–3 in Result section). In parallel, H1, H2, and H13 were also successfully cultured using rice grains as nutrients, with 5–7 rice grains per dish (Figure 1).

Placozoans are transparent, but their color could be changed depending on the algae they are feeding on. For example, light-brownish color occurs with *T. marina* as a food source, medium brownish coloration was observed in animals fed on diatoms (*Entomoneis paludosa* (WoRMS Aphia, ID 163646)), or pinkish colors were seen when animals were fed on cyanobacteria. Pinkish coloration might be due to the accumulation of phycobilins from cyanobacteria, red algae, and cryptophytes.

Under long-term culturing, animals were divided every 1–2 days without signs of sexual reproduction (Malakhov, 1990; Zuccolotto-Arellano and Cuervo-González, 2020).

In contrast to other placozoans, the H4 haplotype (or *Hoilungia* sp.) could be successfully cultured at 28°C using a green algae *T. marina* mixture and two cyanobacteria *Spirulina versicolor* and *Leptolyngbya ectocarpi* (see also Okshtein, 1987). However, if the H4 was maintained on *T. marina* only, the population growth was significantly declined.

### Cryofixation for Transmission Electron Microscopy

Animals were placed in cryo capsules 100 µm deep and 6 mm diameter in ASW. After specimens adhered, the media was replaced with 20% bovine serum albumin solution in ASW. Animals were frozen with a High-Pressure Freezer for Cryofixation (Leica EM HPM 100). After fixation, animals were embedded in epoxy resin (EMS, Hatfield, UK). Ultrathin (65 nm) serial sections were made using Leica EM UC7 ultramicrotome. Sections were stained in uranyl acetate and lead citrate (Reynolds, 1963) and studied using JEOL JEM-2100 and JEOL JEM-1400 (JEOL Ltd., Tokyo, Japan) transmission electron microscopes with Gatan Ultrascan 4000 (Gatan Inc., Pleasanton, CA) and Olympus-SIS Veleta (Olympus Soft Imaging Solutions, Hamburg, Germany) transmission electron microscopy (TEM) cameras. TEM studies were done at the Research center “Molecular and Cell Technologies” (Saint Petersburg State University).

### Laser Scanning Microscopy

Swarmer-like structures and “spheres” were transferred from cultivation dishes to sterile Petri dishes using a glass Pasteur pipette. Individual animals were allowed to settle on the bottom overnight. Fixation was achieved by gently adding 4% paraformaldehyde in 3.5% Red Sea salt (at room temperature) and maintained at 4°C for 1 h. Next, preparations were washed in phosphate buffer solution (0.1 M, pH = 7.4, 1% Tween-20) three times (20 min) and mounted on a slide using Prolong gold antifade reagent with DAPI, and stored in the dark at 4°C. The samples were examined using Zeiss LSM 710 confocal laser scanning microscope with a Plan-Apochromat 63x/1.40 Oil DIC M27 immersion lens (Zeiss, Germany). The images were obtained using the ZEN software package (black and blue edition) (Zeiss, Germany). Image processing was carried out using ZEN (blue edition), Imaris, ImageJ software.

### Statistical Analysis

For population growth rate (PGR) experiments, we cultured axenic lines of H1, H2, H4, and H13 at constant temperature (24°C) and natural light in environmental chambers for 13 experimental days. PGR, locomotion, regeneration analysis, number of animals and occurrences of aggregates were monitored daily at the same time and calculated using standard statistical (*t*-test) and heatmap packages in R. We use triplicates for population growth rates; see additional details in Result section (for original datasets and all details for row data, see Supplementary Tables S1, S2, Supplementary Doc S3, Supplementary Figure S6). We observed exponential-type growth rates for H1 (Avg = 477), H2 (Avg = 312), and H13 (Avg = 232) haplotypes in triplicate experimental groups.

An average (Avg) daily growth of the culture was calculated as numbers of animals per dish in each independent replicate:

$$\nu_{absi}=\frac{n_{i}-n_{i-1}}{t},$$

*n*—numbers of animals, *i*—day for which the speed is estimated $\left(i=\bar{2,11\left(13\right)}\right)$ , *t*—1 day.

The average values for 2–11 and 13 days were calculated from three replicates, and the confidence intervals for the average values. For each independent replication, the relative rate of population growth was presented as:

$$\frac{\frac{\nu_{absi}}{n_{i-1}}\cdot 100\%}{t}$$

We use the Student’s *t*-test for each analyzed parameter (*α* < 0.05 and α < 0.01).

### Treatment of *T. adhaerens* With Antibiotics

*Trichoplax* might contain potentially symbiotic bacteria in fiber cells (Driscoll et al., 2013; Kamm et al., 2019a). To control levels of potential bacterial endosymbionts, we used treatment with different antibiotics (ampicillin (5 μg/ml), doxycycline (1.25 μg/ml), ciprofloxacin (7 μg/ml), and rifampicin (1.25 μg/ml).

Total DNA from individual animals was extracted using a silica-based DiaTom DNAprep 100 kit (Isogene, Moscow, Russia) according to the manufacturer’s protocol. Amplification was performed using EncycloPlus PCR kit (Evrogen, Moscow, Russia) using the following program: 95°C—3 min, 35 cycles of PCR (95°C—20 s, 50°C—20 s, 72°C—1 min), and 72°C—5 min. We have used universal forward primer 27F (AGA GTT TGA TCM TGG CTC AG) and specific reverse primer 449R (ACC GTC ATT ATC TTC YCC AC). The reverse primer was designed against 16S RNA of *Rickettsia belli* (NR_074484.2) and sequences from *Trichoplax* DNA found through NCBI Trace Archive Blast using NR_074484.2 as the query. After 6 months of ampicillin treatment, the *Rickettsia* were not detected. Other antibiotics were less effective (Supplementary Figure S5, see Supplement).

## Results

### Three States of Long-Term Culturing and Feeding in Placozoa

Analysis of growth and behavioral patterns during the long-term culturing allowed us to distinguish three distinct conditions shared across placozoans.

#### Optimized Culture Conditions

This first mode describes a population with a stable growth rate on the established algal mat (∼6.5 × 10^6^ cells of *T. marina* per 1 μL, added once a week) or rice grains (4–5 grains in one Petri dish, diameter 9 cm), and refreshing liquid medium once in 7–10 days. We observed regular fission of placozoans, at average once 1–2 days, during a few months (Figure 3, Mode 1, Supplementary Figure S6). Here, we transferred an excess of animals to other dishes maintaining about 500 individuals per dish. This culturing allows keeping stable populations of placozoans for a long time (from a few months to 2–5 years).

**FIGURE 2:**
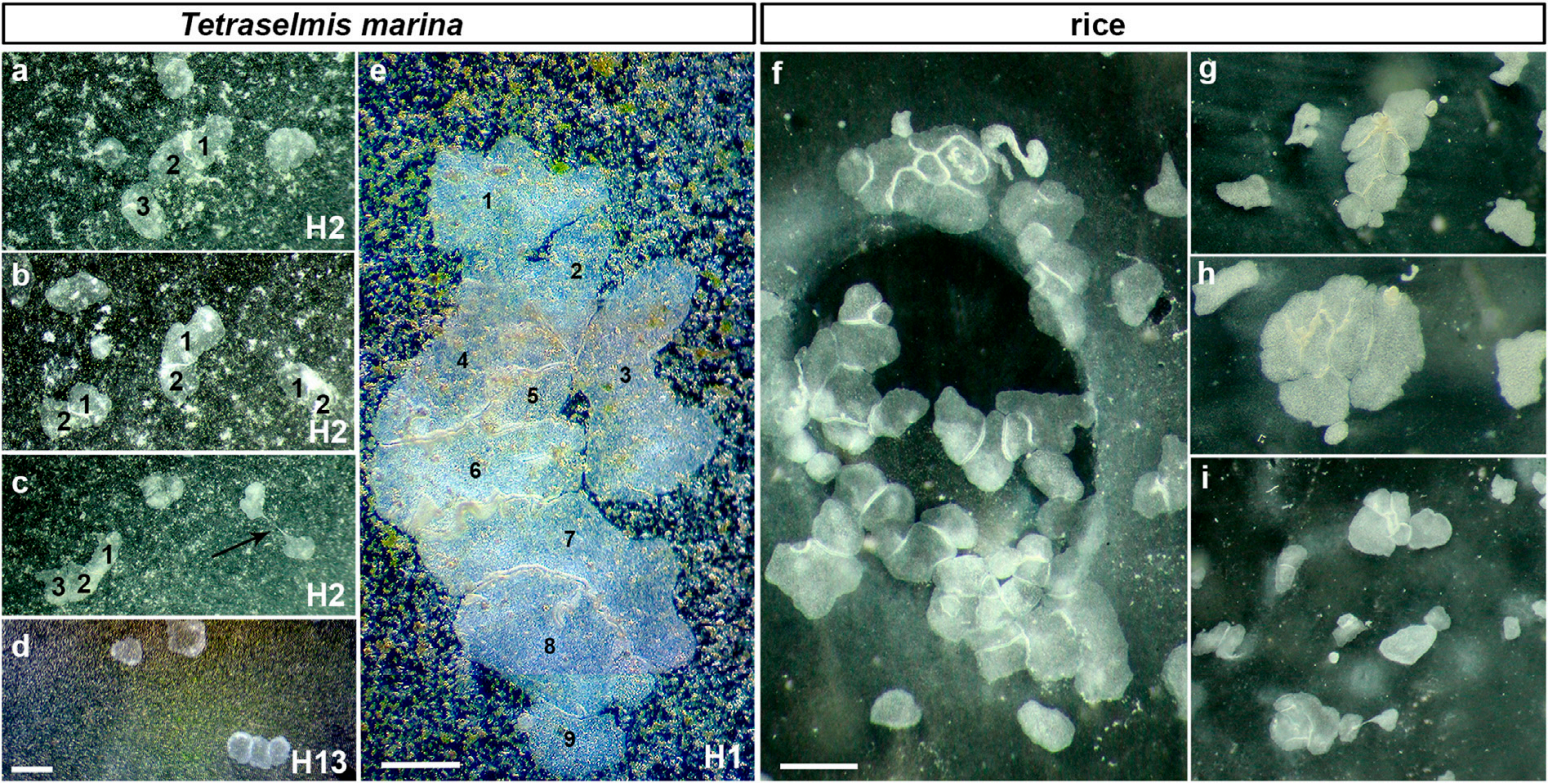
Social feeding behavior during long-term culturing on the dense algal (*Tetraselmis marina* box) and rice (for H1-f) substrates (illustrated examples). The aggregation of animals depended upon the density of the substrates (see mode #3 in the text and Figure 3). This behavioral pattern was observed for all haplotypes (H1, H2, H13—in Petri dishes). The arrow in c indicates a fission process. Aggregates often included 2–15 individuals. For example, there were aggregates of 2-3 animals in H2 **(A–C)** on *Tetraselmis marina*, and 9 H1 individuals **(E)** at the same substrate; but H2 often forms aggregates for 10–15 animals around rice grains **(G–I)**. pH = 8.2. Scale bar: **(A–D)**—100 μm, **(E)**—1 mm, **(F–I)**—500 µm.

Figures 1, 2 show a diversity of canonical placozoans body shapes (Figures 1A–D). However, unusual morphologies were also observed in all haplotypes. For example, we noted animals with a “hole” in the middle of the body (Figure 1E), elongated “pseudopodia”-like structures (Supplementary Figure S3, Supplementary Figure S3), or numerous small ovoid formations in the rim area (Figure 1G).

#### Depleted Food Substrate

**FIGURE 3:**
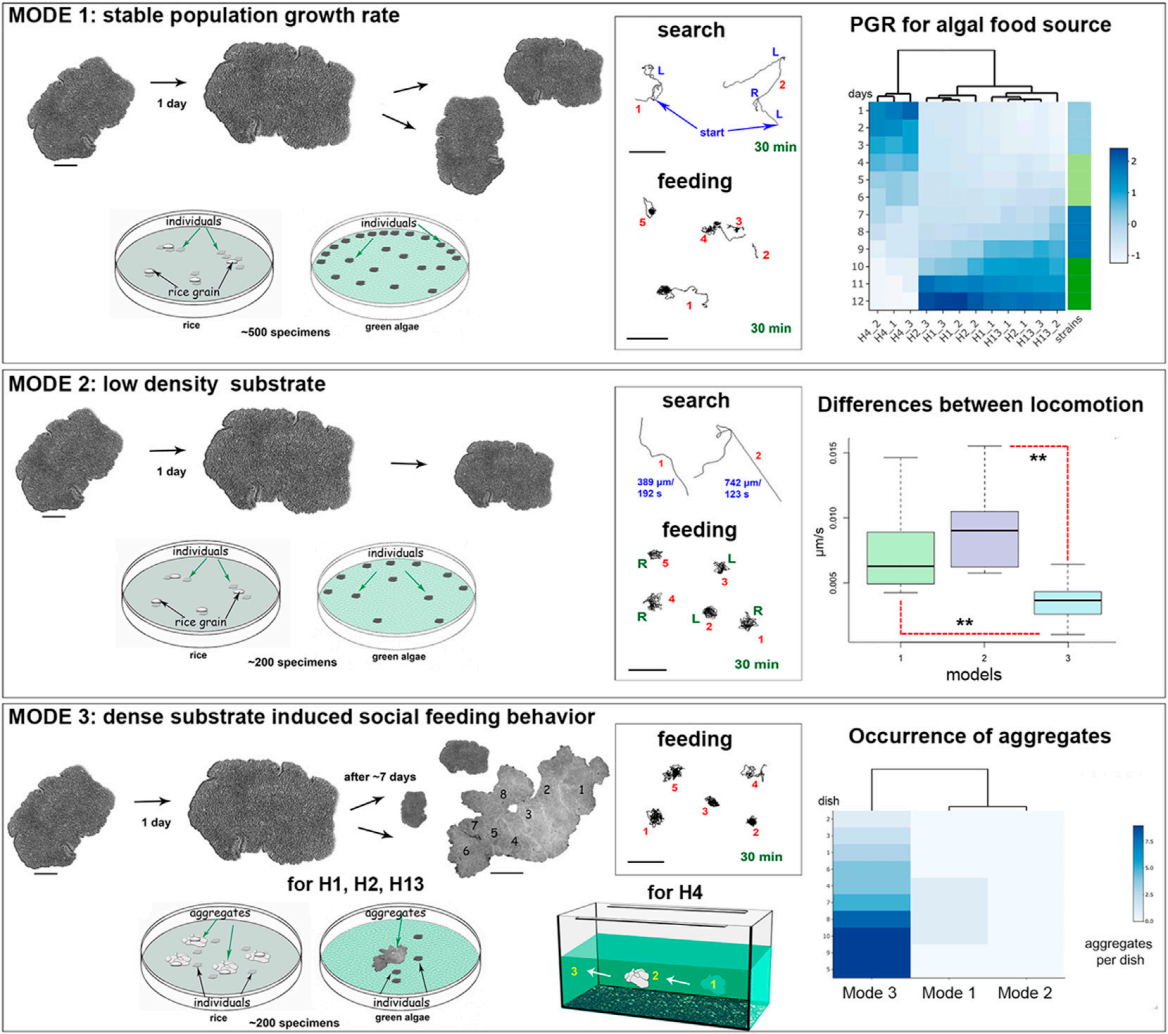
Three separate model schemes of long-term culturing/aging of Placozoa. Mode 1—optimized conditions; Mode 2—low and Mode 3—high densities of the green alga mat (*Tetraselmis marina*) or rice grains; see text for details. Mode 1: Optimized culture conditions with dispersal animals and their moderate concentration on feeding substrates. The middle diagram shows examples of exploratory and feeding locomotion for five animals (numbers) within 30 min. The right diagram shows the representative dynamics of the population growth rate (PGR) for all haplotypes (H1, H2, H4, and H13). 3 separate dishes were used for each haplotype (e.g., H4_1, H4_2, H4_3, etc.), starting with 10 animals per dish. All datasets were normalized to absorb the variation between columns for all four haplotypes of Placozoa. Under these conditions, animals steadily increase their body surface area and have vegetative (non-sexual) reproduction by fission. Mode 2: Low-density substrate. Limit of food source led to decreasing of animal sizes and numbers of animals in culture dishes. Mode 3: High-density substrate. There is both increasing in animal sizes and the aggregation of 2–15 individuals around rice grains or on the dense algal mat. The heat diagram on the right shows the predominant occurrence of aggregates compared to Mode 1 (no aggregates were observed on low-density substrates in Mode 2). H4 expressed the same behavior patterns on the walls of 20L aquarium, where individuals within the aggregate could move together (1-2-3, arrows). However, most animals stay at the substrate (central diagram) with significantly reduced overall locomotion during the feeding, as indicated in the right middle diagram. Each set of video images (Mode 1–3) was analyzed using ImageJ (NIH), calculating for velocity, animal area, and perimeter (*n* = 3–6), as was reported elsewhere (Romanova et al., 2020a). Difference between locomotion in Modes 1 and 3: *p*-value is 0.003002; between Modes 2 and 3: *p*-value is 0.000072 (unpaired Student’s test). Scale bars: for individual animals—200 μm; for the aggregate in Mode 3—1 mm; for all locomotory tracks—200 µm.

If no additional food source was added within 2–3 weeks, the biofilm of microalgae became thinner or depleted. When the layer of microalgae became less than 4.2 × 10^5^ cells/μL, we observed a 1.5-2 fold reduction in animals’ surface areas in all tested haplotypes (H1, H2, and H13), and the population size decreased from ∼500 to ∼200 animals per one cultivation dish (Figure 3, Mode 2). Under these conditions, the animals were concentrated in the densest areas of the algal substrate. In 4–5 weeks, several percent of placozoans formed unusual spherical structures described in *Spherical Formations and Systemic Immune Response*.

#### High Density of Food Substrate and “Social” Behavior

The third mode of culturing was observed on dense substrates such as 3–4-layer algal biofilm with 8 × 10^8^ cell/µL of suspension or 7–8 rice grains (per Petri dish, diameter 9 cm for H1, H2, and H13). Placozoans often formed clusters consisting of multiple animals within a few days on abundant food sources (Figure 2). These aggregates of 2–15 animals have been described as “social” behavior (Okshtein, 1987; Fortunato and Aktipis, 2019). These conditions also induced social feeding patterns in the 20 L aquaria system for *Hoilungia* sp. (H4 haplotype, Figure 3, Mode 3).

This collective behavior differed from typical alterations of search/exploratory and feeding cycles observed in sparked individuals under conditions with limited food sources (Modes 1 and 2, Figure 3). When animals were feeding, they usually stayed on the food substrate or rotated for ∼15–30 min within a small region, comparable to their body length (Figure 3).

## Life Strategies

### Vegetative (Asexual) Reproduction

**FIGURE 4:**
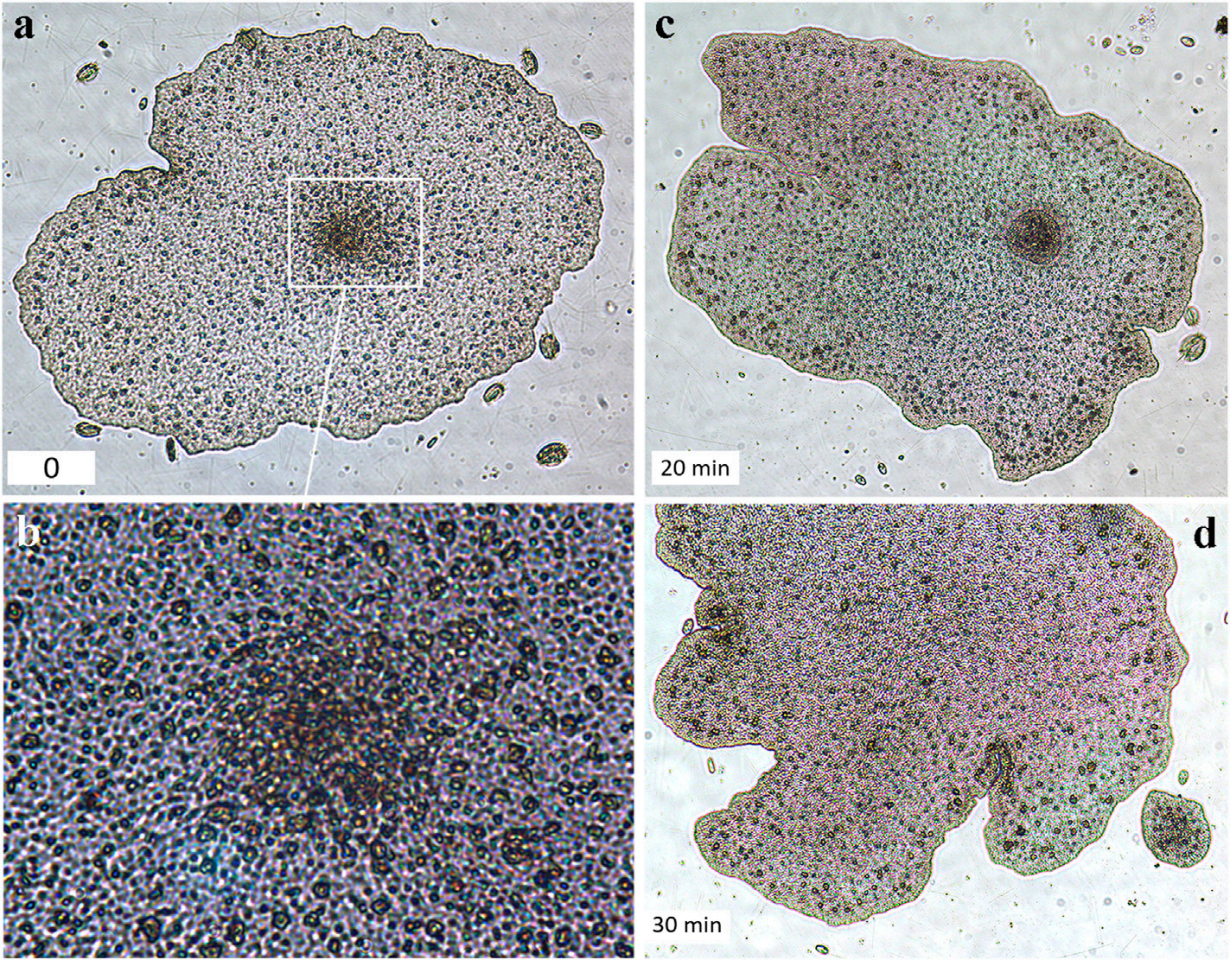
Swarmer-like forms were formed at the lower (substrate-facing) side of the “mother”-animal (H1 haplotype, *T. adhaerens*). **(A,C,D)**—A unique illustrated example of the swarmer formation’s time course (bottom left corners indicate time intervals in minutes). **(A)**. The formation of a higher density cell region in the middle part of the mother animal (white square outline, and the higher magnification of the same region in **(B,C)**. The formation of the swarmer and its separation from the mother animal **(D)**.

**FIGURE 5:**
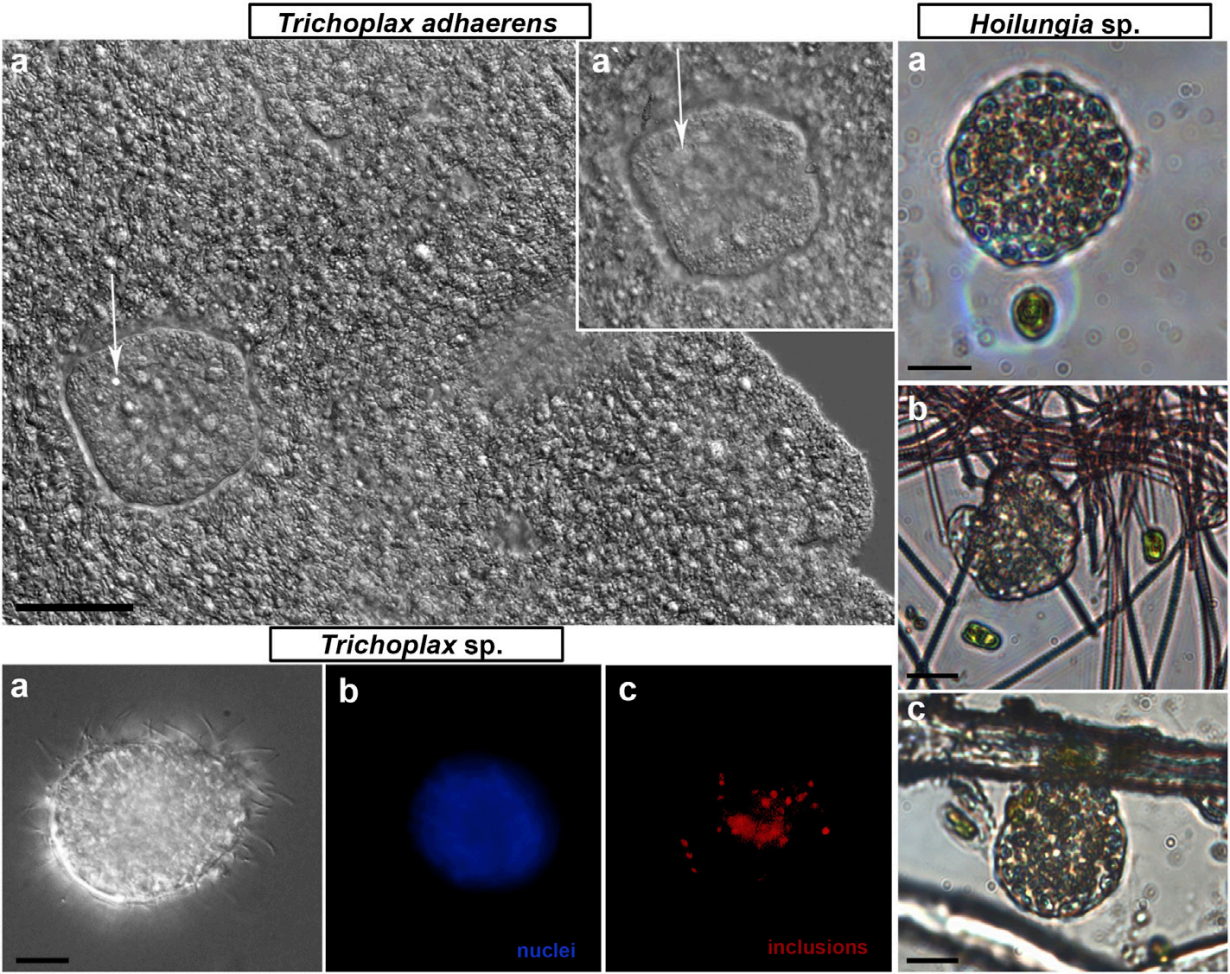
“Swarmers” were formed in the long-term culture in every studied species of Placozoa. “Swarmers” are small (15–30 µm diameter) juvenile animals (white arrows in *T. adhaerens* box, **(A–A′)**—different Z-layer). “Swarmers” expressed coordinated exploratory and feeding behaviors (*Hoilungia* sp. box, H4 haplotype, three illustrative images of different juvenile animals). *Trichoplax* sp. box: (a)—DIC view of the solid swarmer-like animal; (b)—high density of nuclei in the center; (c)—autofluorescence. Scale bar: 10 µm.

Long-term culturing provided additional insights into the life-history strategies of Placozoa. In addition to the fission, the formation of smaller daughter animals or “swarmers” had been described in *Trichoplax adhaerens*, and swarmers were reportedly derived from the upper epithelium (Thiemann and Ruthmann, 1990; Thiemann and Ruthmann, 1991). Here, we observed the development of swarmer-like forms in all haplotypes studied (H1, H2, H4, and H13; Figures 4, 5), suggesting that it is an essential part of adaptive strategies for Placozoa [see Supplementary Video S4 for *Trichoplax* sp. (H2), and Supplementary Video S5, S6 for *Hoilungia* (H4 haplotype)].

The formation of “swarmers” occurred spontaneously (in about 2 or 5 weeks from a cultivation start) both on algal biofilms and rice. But we noted that swarmer-like forms could be formed at the lower, substrate-facing side (Figure 4, Supplementary Video S3), with a significantly greater cell-type diversity than in the upper layer (Smith et al., 2014; Mayorova et al., 2019; Romanova et al., 2021). Therefore, the formation of swarmer-type forms from the lower layer might be facilitated by the preexisting heterogeneity of cell types in this region. After physical separation, these progeny could be temporally located under the “mother” animal (Supplementary Video S3), moving together on substrates or biofilms.

### Regeneration as a Part of Adaptive Life Strategies in Placozoans

An overpopulated/fast-growing culture with a high density of placozoans (over 500–700 animals per 1 Petri dish 9 cm in diameter) often contains many floating individuals or individuals on walls, which are frequently aggregated under the surface film (Supplementary Figure S2). The animals could be raptured as a result of contact with air and/or other mechanical damage. Nevertheless, such fragmentation often led to regeneration, which we consider an essential part of life strategy in placozoans.

**FIGURE 6:**
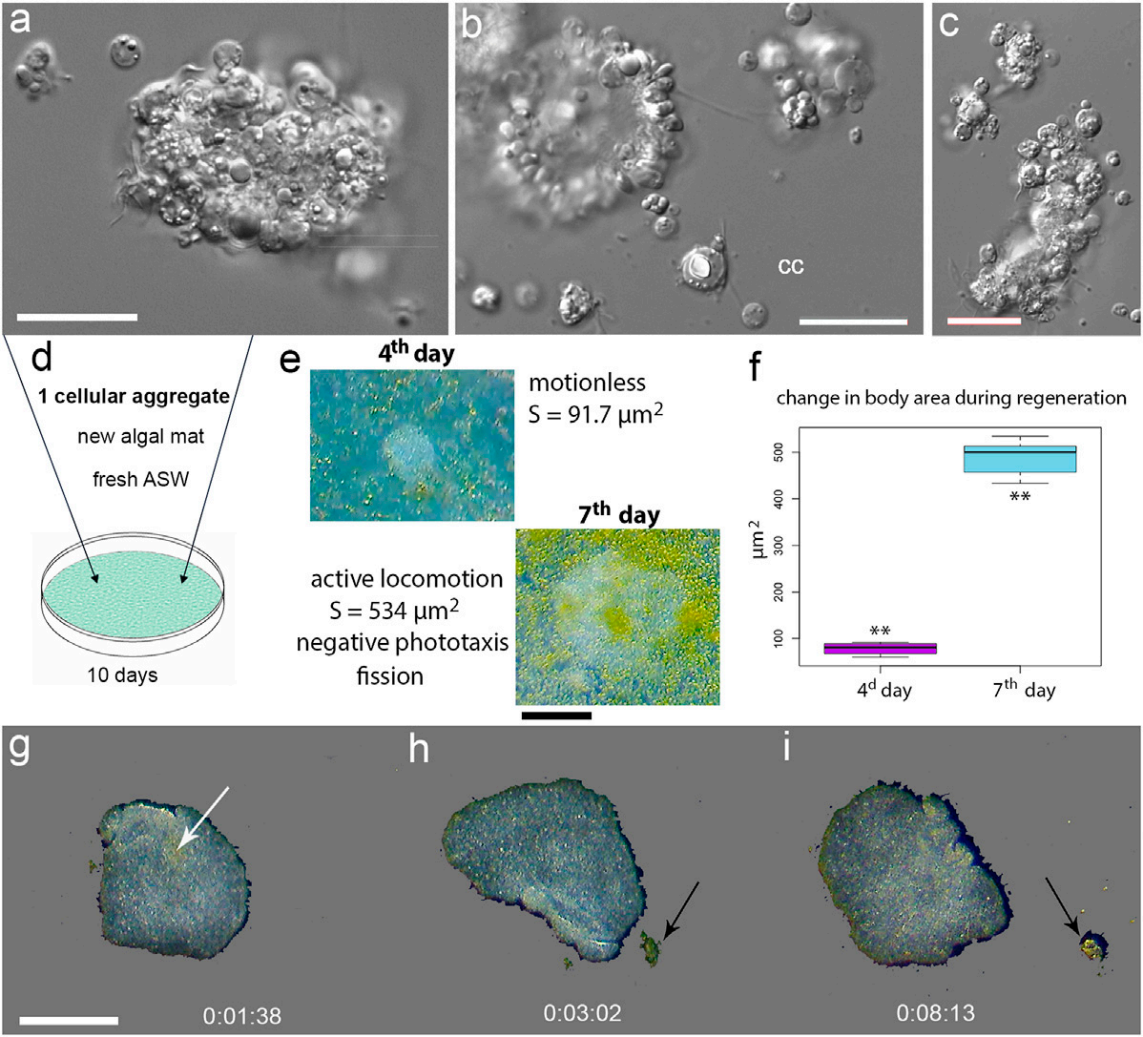
Regeneration in Placozoa. **(A,B,C)**—Illustrative examples of small cellular aggregates from *T. adhaerens*. Aggregates consist of ciliated epithelial cells, lipophil, crystal, and fiber cells, which were identified based on their distinct morphology. cc-isolated crystal cell. **(D)**—Placement of a single aggregate in a new culture cell with fresh ASW and algal mat. **(E)**—4th and 7th day of regeneration with calculated surface area (S) of a newly formed animal. The ciliated locomotion, negative phototaxis and fission started on the 7th day of regeneration. **(F)**—increasing surface areas occurred from 4th to 7th day. The t-value: −23.48979. The *p*-value: < 0.00001. **(G–I)**—time-lapse images after splitting an animal into two parts; locomotion and feeding continued (see text and Supplementary Video S7). Arrows indicate a cluster of algae. Scale bar: **(A,B,C)**—20 μm, **(E)**—200 μm, **(G–I)**—500 µm.

We investigated the regeneration in model experiments (using H1 and H2 haplotypes, 1–2 mm in size). Individuals were damaged in two ways: mechanical injury by pipetting and by cutting animals with a scalpel (into two parts). The former protocol allows obtaining small cell aggregates (∼20–30 cells) placed in Petri dishes with biofilms of *Tetraselmis marina*. The regeneration process lasted approximately 7–10 days. The first stage of recovery was an increase in cell numbers within the aggregates, which were immobile (Figure 6). Notably, intact placozoans had a negative phototaxis (animals moved to the darkened areas of experimental Petri dishes, see Supplementary Figure S1). However, at the early stages of regeneration, *Trichoplax* aggregates did not respond to changes in light intensity, remaining motionless. After the 4th day, locomotion was restored (Figure 6).

On the 7th day, original aggregates became small individual animals with active locomotion and feeding behaviors as well as capable of fission and negative phototaxis. Interestingly, if dissociated cells and aggregates were transferred to Petri dishes without a food source, then aggregates were lysed after 2–3 days.

After dissection individuals into two parts, we observed a slight contraction of animals. Still, within a few minutes, animals curled up, closed the wound, and moved without detectable changes in their locomotion patterns (Figure 6, Supplementary Video S7).

### Spherical Formations and Systemic Immune Response

**FIGURE 7:**
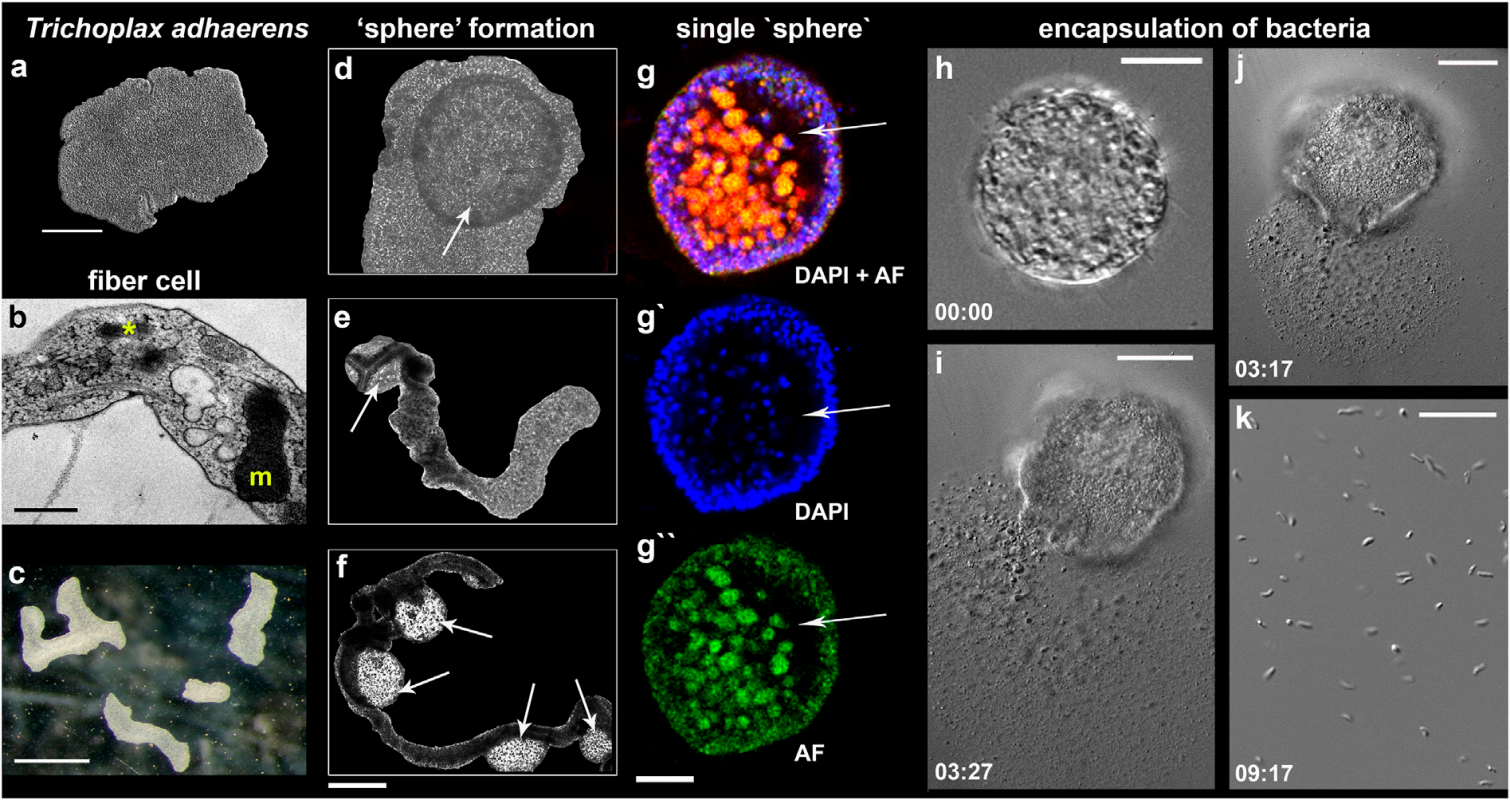
“Sphere”-type formations in *Trichoplax adhaerens*. **(A–C)** control animals under optimal culture conditions (mode #1, see text and Figure 3); **(B)**—Transmission electron microscopy (TEM) image of the fiber cell with a bacterium (asterisk) and a large mitochondrial complex (m). **(D–H)**–Formation of spheres (arrows) from the upper/dorsal epithelium. **(D)**—disk-like animal; **(E,F)** -animals with elongated bodies; **(E)**—one “sphere” (arrow); **(D)**—four “spheres” (arrows). (**G–G″)**—Separated spheres with internal cavities (arrows); Nuclear DAPI staining—blue [excited by the violet (∼405 nm) laser with blue/cyan filter (∼460–470 nm)]; Autofluorescence (AF)—green (excitation 490 nm and emission 516 nm). **(I–K)—**Spherical formations encapsulate bacteria inside; **(I–J)**—the damage of the sphere’s surface by laser released numerous bacteria (magnified in **(K)**; see text for details). Time intervals following the laser-induced injury are indicated in the left corners of each image. Scale: a 200 μm, **(B)**—500 nm, **(C)**—1 mm, (**G–G″)**—400 μm, **(G–I)**—20 μm, **(K)**—10 µm.

**FIGURE 8:**
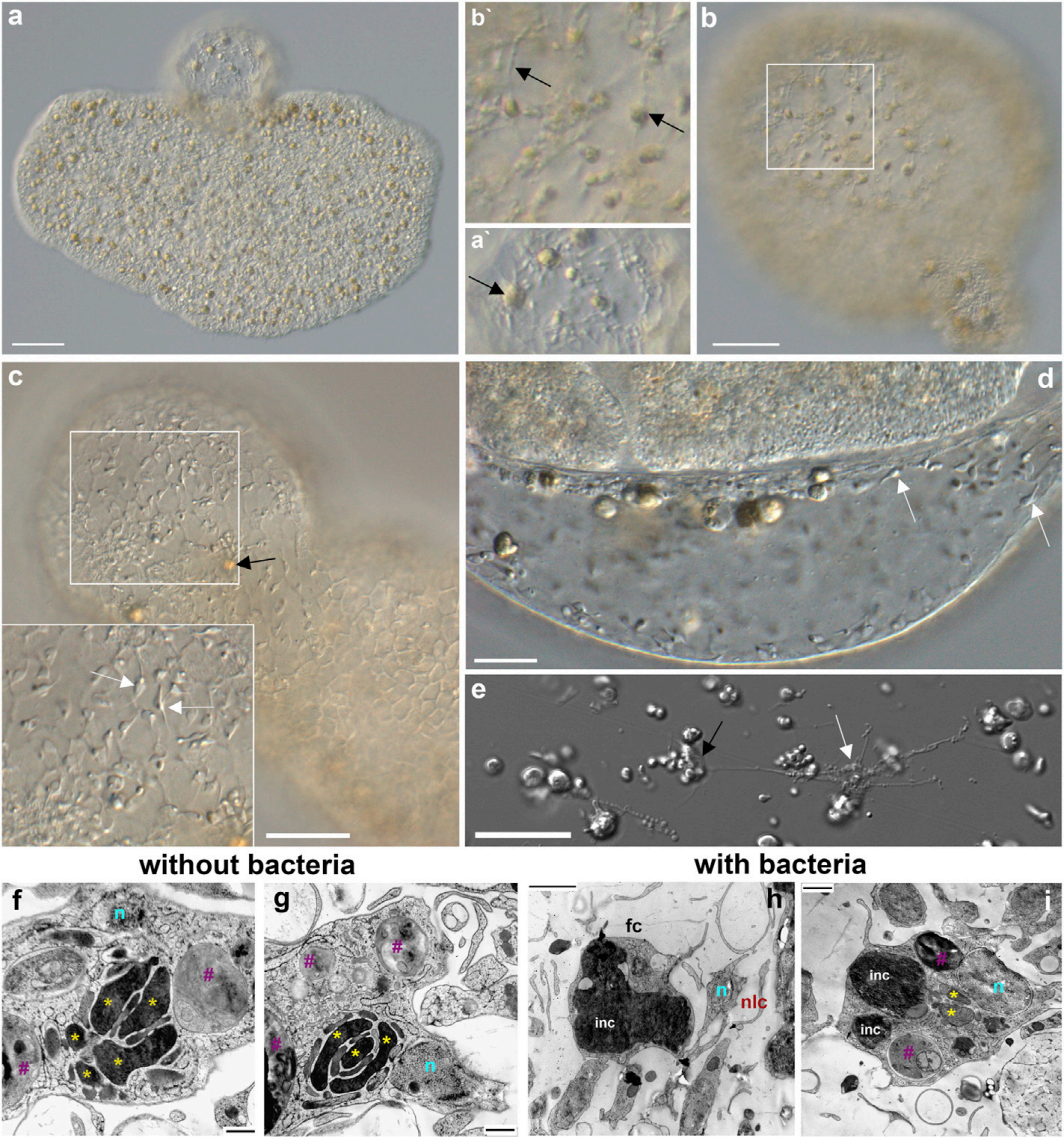
Fiber cells in spherical structures **(A–C)** and their microanatomy **(F–I)**. Both fiber cells (black arrows) and smaller neuroid-like cells (white arrows) are present in “spheres’”. **(A)**—*Trichoplax adhaerens* with a sphere on the upper side. **A′**– a part of the sphere with two fiber cells (arrow). **(C,D,E)**—smaller neuroid-like cells (white arrows) with elongated processes **(E)** that can form connections among themselves and fiber (black arrow) cells (Romanova et al., 2021). Both cell types are located in the middle layer of placozoans **(B/B′)**. **(F–I)**—Transmission electron microscopy (TEM) of the fiber cells in *Rickettsia*-free *Trichoplax* (ampicillin-treated for 12 months) and control animals with endosymbiotic bacteria (see also Figure 7B). Of note, fiber cells in ampicillin-treated populations of placozoans had more elaborate mitochondrial clusters and clear inclusions. In contrast, in animals with bacteria, fiber cells possessed large dark (by TEM) inclusions (light microscopy also shows brownish inclusions—black arrows in **A–C**). Yellow asterisks (*) - mitochondria, n—nuclei, purple #—clear inclusions, inc—dark inclusions, fc—fiber cells, nlc—neuroid-like cells.

In 4–5 weeks (Mode 2 of culturing with depleting food source), some animals started developing specific spheroid structures (Figures 7, 8, Supplementary Video S8–S12). The formation of these “spheres” occurred randomly in 2–5% of individuals, and data reported below are based on observations of about one hundred animals with such structures. We hypothesize that “spheres” can be viewed as a component of innate immune (e.g., bacterial infection) responses; and separated three stages of this process.

The first stage was an apparent lengthening of body shape (Figure 7C) and inability to fission (Figures 7C–F). This phenomenon was observed during the aging of populations, which occurred without systematic refreshing of ASW. When ASW was replaced within 2–3 days, we restored healthy populations of placozoans without any noticeable morphological changes compared to control individuals (as in Mode 1).

The second stage: the upper epithelium begins to exfoliate (Figures 7D–F, Supplementary Figure S7), and spherical formations become visible. We could observe elongated animals with one “sphere” (Figure 7E) or several spheres (Figure 7F), as well as rounded animals with one “sphere” (Figure 7D).

The third stage was a separation of the spherical structures from a mother animal (Figures 7G–7G). “Spheres” consisted of upper epithelium, fiber cells, and, probably, “shiny spheres” cells (with large lipophilic inclusions). Light microscopic analysis revealed cavities inside “spheres” (Figures 7G–7G). We damaged the surface tissue of the “sphere” with laser beams (using confocal microscopy), which released numerous bacteria-sized particles (unknown identity, Figures 7H–K), suggesting that these spherical formations could encapsulate bacteria inside (Supplementary Video S13, Supplementary Figure S8).

#### Reversed Nature of “Spheres”

When we transferred 28 already separated “spheres” (Supplementary Figure S4) to new Petri dishes with algal mats, then within 3–5 days, we observed the restoration of classical placozoan bodyplan. During the next few days, stable populations of placozoans could be established. In contrast, when we placed 28 control spheres in sterile Petri dishes without algal mats, all “spheres” were degraded within 2–5 days.

Fiber cells are capable of phagocytosis (Thiemann and Ruthmann, 1990; Jacob et al., 2004; Moroz and Romanova, 2021) and contain bacterial cells (Guidi et al., 2011; Gruber-Vodicka et al., 2019; Kamm et al., 2019a; Romanova et al., 2021). We also confirmed that bacteria were localized in fiber cells of H1, H4, and H13 (Figure 8B). It was hypothesized that bacteria could be endosymbionts (Kamm et al., 2019a; Gruber-Vodicka et al., 2019). Moreover, the ultrastructural analysis of fiber cells suggested the engulfment of bacteria (Figure 7B) by the endoplasmic reticulum, which can also be viewed as a stage of intracellular phagocytosis (Figure 8B).

The treatment of *Trichoplax* with ampicillin eliminated potential bacteria from placozoans (Supplementary Figure S5) and their fiber cells (Figures 8F–I). Furthermore, ampicillin prevented the formation of “spheres” in bacteria-free culture: none were seen during 12 months of cultivating *Rickettsia*-free animals (over 10,000 animals, Supplementary Figure S5). Of note, in our culture, animals lived in the presence of ampicillin for more than 4 years without bacteria.

The fiber cell type contains the massive mitochondrial cluster (Figures 7B, 8F,G; Grell et al., 1991; Smith et al., 2014; Mayorova et al., 2018; Romanova et al., 2021, Figure 5) as a reporter of high energy production. Ampicillin-treated, *Rickettsia*-free animals had additional morphologic features such as numerous small and clear inclusions inside fiber cells (Figures 8F,G). The control group with bacteria has one or two large inclusions (Figures 8H,I) with a less visible mitochondrial cluster. The functional significance of these ultrastructural changes is unclear.

## Discussion

**FIGURE 9:**
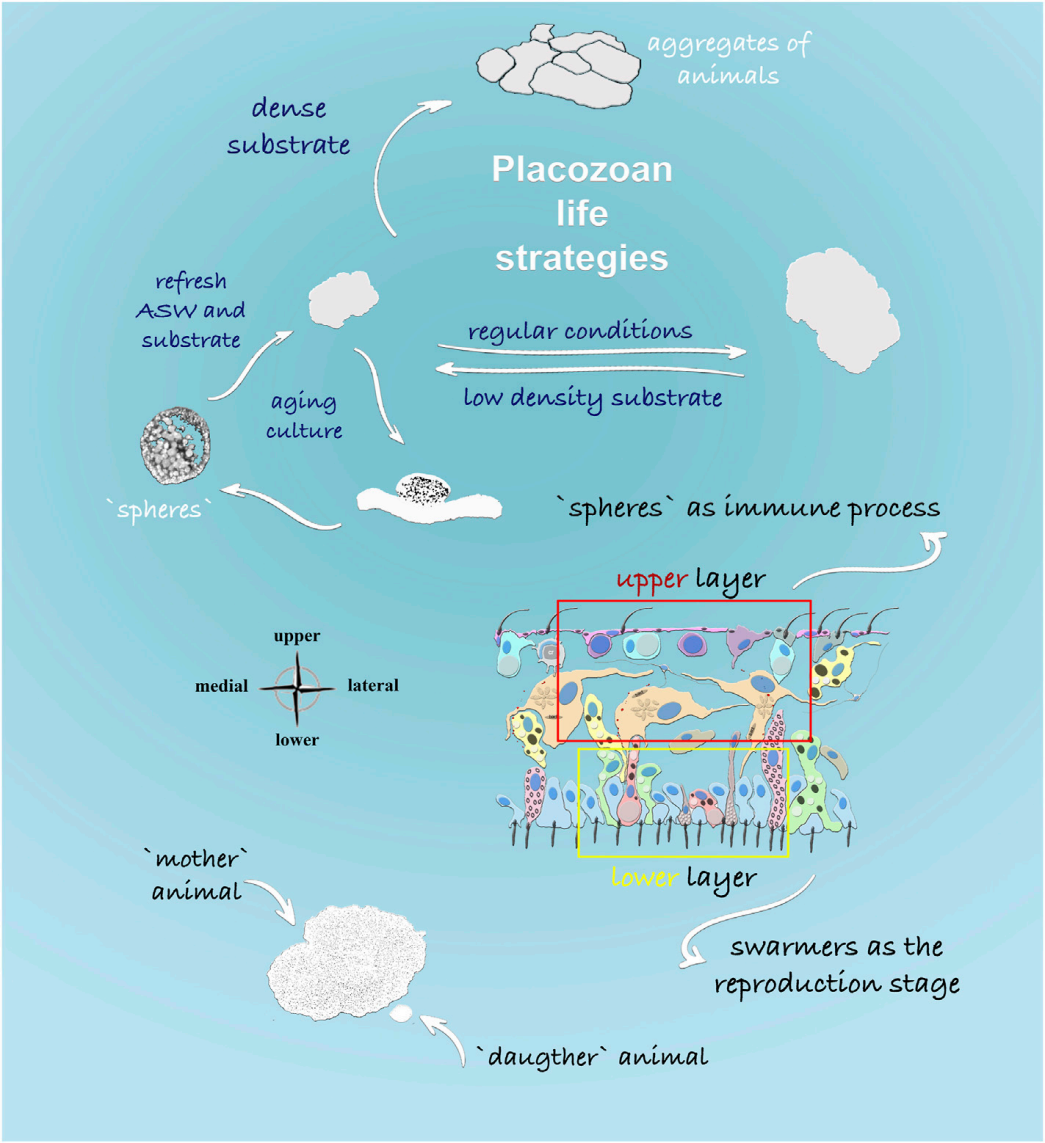
Life Strategies in Placozoa: Schematic representation of feeding and reproductive stages described in this study. The density of food substrate predominantly determines formations of different morphological stages. Dense algal substrate led to the formation of aggregates from multiple animals (“social” feeding behavior). In aging culture, the formation of specialized spherical structures was observed from the upper cell layer of placozoans, and it was shown that spheres could harbor multiple bacteria. In contrast, the formation of small juvenile animals or “swarmers” might have different etiology and development from the lower cell layer, as shown in the cross-section of *Trichoplax*.

Grazing on algal and bacterial mats might be an ancestral feeding mode in early Precambrian animals (Rozhnov, 2009). And placozoans may have preserved this evolutionarily conserved adaptation from Ediacaran animals (Sperling and Vinther, 2010). Under this scenario, we view the long-term culturing of placozoans as an essential paradigm to study interactions among relatively small numbers of cell types for integration of morphogenesis and reproduction, immunity, and behaviors. Figure 9 summarizes the complementary life-history strategies present in at least four species of Placozoa (=H1, H2, H4, and H13 haplotypes).

The directional (ciliated) locomotion in placozoans depends upon distributions of food sources, which differentially alternate exploratory and feeding patterns. Dense biofilms triggered social-type interactions and elementary cooperation, whereas limited food supplies, stress, and aging triggered systemic immune and morphogenic responses as well as alternative modes of reproduction.

Placozoans are virtually immortal with dominant clonal reproduction strategies (see Schierwater et al., 2021 and below). Furthermore, our data with small cell aggregates (*Regeneration as a Part of Adaptive Life Strategies in Placozoans*) suggest that regenerative responses might also be part of adaptive reproduction mechanisms.

Placozoans have two classical types of non-sexual reproduction: 1) fragmentation into two daughter animals or fission and 2) formation of swarmers (Thiemann and Ruthmann, 1988; Thiemann and Ruthmann, 1990; Thiemann and Ruthmann, 1991; Seravin and Gudkov, 2005a, b)—juvenile animals with small body size (20–30 µm). Thiemann and Ruthmann showed that the “budding” of a daughter animal started from the dorsal side for 24 h, and a released swarmer had all four morphologically defined cell types at that time (Thiemann and Ruthmann, 1988; Thiemann and Ruthmann, 1990; Thiemann and Ruthmann, 1991; Ivanov et al., 1980b). Here, we observed that the formation of swarmer-like juvenile animals could also occur from the lower layer in all placozoans tested here. This type of arrangement might have some rationale: the lower epithelium consists of a greater diversity of cell types (compared to the upper layer) such as epithelial, lipophil, and gland cells with various subtypes (e.g., Smith et al., 2014; Mayorova et al., 2019; Romanova et al., 2021; Prakash et al., 2021).

### The Emerging Diversity of Life Forms in Placozoa

In addition to classical flat, disk-like animals, the earlier literature suggests that at least six different spherical morphological forms occur in Placozoa. Thiemann and Ruthmann (1988); Thiemann and Ruthmann (1990); Thiemann and Ruthmann (1991) used electron microscopy to characterize the budding process in *Trichoplax adhaerens*. They provided morphological descriptions of “swarmers” and “spherical forms” as well as the distributions of the different cell types within these structures. The spherical forms were named as follows: 1) Moribund or non-viable spherical forms (degenerative, non-reproductive phase) according to Grell, 1971; 2) Hollow spheres, type A, which cannot be transformed to flat animals (Thiemann and Ruthmann, 1990); 3) Hollow spheres, type B, which can be transformed to flat animals or “big” (40–60 µm) swarmers (Grell and Benwitz, 1974; Ivanov et al., 1980a; Thiemann and Ruthmann, 1988); 4) Solid small (12–20 µm) swarmers-like forms, or solid swarmers, vegetative reproduction stage (Grell and Benwitz, 1971; Thiemann and Ruthmann, 1988; Thiemann and Ruthmann, 1991); they can be similar to small swarmer-type forms derived from the ventral surface and discussed here; 5) Solid spheres without cavities (120–200 µm), which are not connected to vegetative reproduction (Thiemann and Ruthmann, 1990); 6) Dorsal stolons, which form small daughter animals by mechanisms different from fission and swarmers (Thiemann and Ruthmann, 1991).

The exact relationships between classical “swarmers” (Grell and Benwitz, 1974; Ivanov et al., 1980b; Thiemann and Ruthmann, 1988) and other spherical structures described in previous papers and this manuscript are less evident due to the lack of established terminology, cross-referenced microscopic methods, and details. No electron microscopic observations of these budding processes were undertaken in the present study, but light microscopy confirmed the presence of similar main cell types as described before.

In a broad sense, the formation and morphology of most separated spherical structures overlap with the far-reaching definition of swarmers as small vegetative progeny (Thiemann and Ruthmann, 1988; Thiemann and Ruthmann, 1990; Thiemann and Ruthmann, 1991). In more precise terms, some physically separated spheroid forms described here match the definition of “hollow spheres”, type B (Thiemann and Ruthmann, 1988; Thiemann and Ruthmann, 1990; Thiemann and Ruthmann, 1991), and their potential transformations to juvenile animals. Specifically, solid small swarmers-like forms (Grell, 1981; Thiemann and Ruthmann, 1988; Thiemann and Ruthmann, 1991) can be similar to small swarmer-like forms discussed here and derived from the ventral surface. They can be naturally transformed into “canonical” juvenile animals under favorable conditions in both cases. To sum, the differences between the current and earlier observations on swarmer-type forms might be clarified if we consider the dynamic nature of sphere formation (e.g., Supplementary Video S14), which varies depending on the conditions in which animals are cultivated. We observed the enhanced formation of spherical buds in animals maintained with reduced food sources and aging cultures.

### The Roles of Spheres in Innate Immunity Responses

We hypothesize that the spheres could be developmentally linked to the innate immune response, possibly induced by bacteria in aging populations or unhealthy culture conditions. Thus, hollow spheres developed from the upper layer could also be a path to vegetative reproduction under stress conditions. If the microenvironment became more favorable for placozoans, “spheres” can be transformed into swarmer-like forms as pelagic stages of benthic placozoans and eventually juvenile animals.

### The Nature of Bacterial Species and Immunity

Since we did not observe these structures in antibiotic-treated cultures, the budded spheres could be formed as morphological defensive responses to bacterial infection. Bacteria-shaped cells were also released from one of these spherical structures when it was damaged with laser illumination, and we observed their division and labeling with DAPI (Supplementary Figure S8). However, we did not characterize the placozoan microbiome with metagenomic tools and inferred putative bacterial sources as the most likely explanation of morphological observations. Also, we did not know whether these bacteria differed from the previously reported endosymbionts (Kamm et al., 2018; Kamm et al., 2019a). On the other hand, our ultrastructural data confirmed the presence of bacteria in fiber cells of all studied species, and fiber cells could be critical players in the integration of immunity, morphogenesis, defense, and behaviors.

One of the forms of nonspecific defense in invertebrates is the encapsulation of foreign objects, and this process is similar to the systemic sphere formation in Placozoa. We see that opsonization has been observed inside the fiber cells (Figures 7, 8). Plus, the fiber cells can perform the functions of macrophages with a well-developed capacity for phagocytosis (Thiemann and Ruthmann, 1990).

Cellular immunity by phagocytosis is the most ancient and widespread mechanism among basal Metazoa. For example, in sponges and cnidarians, the encapsulation is carried out by amoebocytes (=archeocytes) or collencytes (Musser et al., 2021). Phagocytosis in invertebrates, like in vertebrates, includes several stages: chemotaxis, recognition, attachment of a foreign agent to the phagocyte membrane, intracellular lysis, etc. (Bang, 1975; Lackie, 1980; Bayne, 1990). Due to the limited diversity of cell types, humoral and cellular immune responses could likely be relatively simple in Placozoa (Kamm et al., 2019b; Popgeorgiev et al., 2020).

Chemoattractant/repellents can be signaling molecules from microorganisms or other cell types. Symbiont-host signaling can include changes in nitric oxide gradients (Moroz et al., 2020b) or regional differences in amino acid composition, interconversion of D- and L-forms (Moroz et al., 2020a), the formation of oxygen radicals, which are toxic to bacteria, etc.

Fiber cells are perfectly suitable for the placozoan immune system’s sensors, integrators, and effectors. Fiber cells are located in the middle layer of placozoans with multiple elongated processes, spread around many other cell types, including the crystal cells. Fiber cells have specialized contacts among themselves (Grell and Ruthmann, 1991). A new class of neural-like/stellar-like cells is localized in the vicinity of fiber cells. Together they form a meshwork of cellular processes from the middle layer to the upper and lower layers (Moroz et al., 2021a; Romanova et al., 2021). This “network” can be a functional integrative system sharing some immune and neural features and pools of signaling molecules in multiple microcavities for volume transmission (Moroz et al., 2021b).

We propose that such a placozoan-type integrative system is conceptually similar to the ancestral integration of innate immune and primordial neuroid-like systems, which controlled adaptive stress responses and behavior and regulated morphogenesis and regeneration.

Early (and present) animals strongly depended on the environmental and symbiotic bacteria, and fiber-type cells (or similar/homologous classes of amoebocytes as recently described in sponges - Musser et al., 2021) might be critical elements in the shared evolution of immune and neural systems to integrate both morphogenesis and behaviors (Fields et al., 2020). Here, the defense against bacterial infections can be an inherent part of such integrative ancestral adaptive responses.

Comments added to proof: When this manuscript was under review, Mayorova et al. (2021) provided additional experimental evidence and highlighted the importance of placozoan fiber cells in regeneration, innate immunity, and phagocytosis emphasizing the significance of macrophage-like cells in the evolution of basal animal lineages.

## Data Availability

The original contributions presented in the study are included in the article/Supplementary Material, further inquiries can be directed to the corresponding authors.

## Supplementary Material

Supplementary Figure S1 Negative phototaxis during long-term culturing of *Trichoplax* and *Hoilungia* genera. a. Culture dishes in the environmental chamber; **(B)**, **(C—A)** Petri dish with *T. adhaerens*; animals were concentrated at the darker side of the dish **(B)** vs. more illuminated side **(C)**; **(D)**, **(E—A)** Petri dish with *Hoilungia hongkongensis*; animals were more concentrated at the darker side of the dish **(D)** vs. brighter side **(E)**.

Supplementary Figure 2 Floating individuals in an aging culture of placozoans. The photo in **(A)** shows both single floating animals (red arrows) and aggregations under the water surface (white arrows). **(B)**. An illustrated example from Supplementary Video S2 with a free-floating animal (white arrow); a white circle indicates an animal at the bottom of the cultured dish. Scale bar: A—1 cm, B—1 mm.

Supplementary Figure S3 Dynamic changes in the body shape of *Trichoplax* sp. (H2 haplotype). Note unusual morphological features such as “pseudopodia”-like elongations in a single individual during ∼7 min of time-lapse. Scale bar: 200 μm.

Supplementary Figure S4 Restoration of a disk-shaped morphology from a sphere to a juvenile placozoan *T. adhaerens*. Separation of “spheres”: The photo shows two separate spheres from an animal. Recovery: A particular sphere (∼ 30 μm) was placed in a Petri dish with a freshly prepared algal mat (*T. marina*) and ASW (Mode 1). Note an appearance of flattened/elongated body parts from the sphere (2–6 hrs, first three images). In 24 hrs the sphere was transformed into a disk-shaped animal capable of locomotion. In 2 days, small individuals were capable of fission.

Supplementary Figure S5 Isolation of intracellular bacteria-free *Trichoplax* culture. Table of antibiotics used in experiments. **(A)** Agarose gel electrophoresis of the PCR test of individual *Trichoplax adhaerens* with specific primer for Rickettsiales 16S rRNA (*n* = 5 per antibiotic, total *n* = 20 individuals). After 6 weeks of treatment with three different antibiotics, only a minor decrease in bacterial DNA was detected for doxycyclinum, and no effect for two other antibiotics. Treatments with rifampicinum and ciprofloxacin were terminated after 1.5 and 7 months, respectively, because of inefficiency, while doxycyclinum treatment continued for additional time. **(B)** 6 months after the beginning of the experiment, we started treatment with the fourth antibiotic, ampicillin. Agarose gel electrophoresis with tests for 12 months of doxycyclinum and 6 months of ampicillin treatment. Doxycyclinum culture still contains a minor amount of rickettsial DNA, while the ampicillin line is entirely negative. DNA of 10 animals was pooled for each PCR tube, a total of *n* = 60. The ampicillin-treated line was cultivated with the constant addition of ampicillin for four years by now, and all subsequent tests for rickettsial DNA were negative (not shown). This culture line was used for microscopic imaging shown in Figure 8.

Supplementary Figure S6 Dynamics of the placozoan population growth and pH variations in culture conditions (artificial seawater 35 ppm, pH = 8.0, 24 ± 2°C, and daylight illumination). Two H1 **(A)** or one H2 **(B)** individuals were added into a 9-cm Petri dish with *Tetraselmis marina* biofilm. Two/thirds of the water were replaced on Day 8. The numbers of individuals and the value of the pH in the media were assessed daily (at the same time of the day) for all days. H13 **(C)** population growth was monitored under similar conditions except that the water was changed daily, yielding a constant pH of 8.0. All experiments were done in triplicates (error bars represent standard error of the mean, confidence intervals **p* < 0.05, ***p* < 0.01).

Supplementary Figure S7 Illustrated examples of the “hollow” sphere formation in *Trichoplax adhaerens*.

Supplementary Figure S8
**The** overall architecture of a “spherical structure” **(A, B)**. DAPI labeling (blue) and cellular autofluorescence (green). Total image size by Z: 41.24 µm, including **(A–A′)**—12.37 µm depth, **(B–B′)**—16.49 µm depth. White arrows **(A, B)** indicate two putative bacterial cells in the sphere, which are different from *Trichoplax* cells with a larger nucleus and autofluorescence in the cytoplasm. **(C, D)**–bacteria-shaped particles released after the sphere’s damage by laser (confocal microscopy and DIC). **(C–C′)** shows the same area with DIC and DAPI fluorescence; blue arrows—*Trichoplax* cell; white circles—putative bacteria cells with a distinct rood-type morphology, which was not observed for *Trichoplax*. Scale bar: **(A–A′)**, **(B–B′)**, **(C–C′)**, **(E)**—20 µm, **(D)**–10 µm.

Supplementary Video S1 The unusual morphology—*T. adhaerens* with a “hole” in the middle of the body (see also Figure 1E).

Supplementary Video S2 Floating individual in an aging culture of *T. adhaerens*. The photo in **(A)** shows both single swimming animals (red arrows) and aggregations under the water surface (white arrows).

Supplementary Video S3 After physical separation of a “swarmer,” this juvenile animal could be temporally located under the “mother organism,” moving together on substrates (*T. adhaerens*).

Supplementary Video S4 A swarmer of *Trichoplax* sp. (H2 haplotype).

Supplementary Video S5 A swarmer *Hoilungia* sp. (H4 haplotype).

Supplementary Video S6 A swarmer *Hoilungia* sp. (H4 haplotype).

Supplementary Video S7 Locomotion of *Trichoplax adhaerens* on the clean glass before and after splitting the animal by a thin steel needle. 5X magnification, 250X time-lapse. The animal was cut at the site marked by a white circle and letter R. Tracks of the two halves are displayed as red and yellow circles. Tracks of the second animal halves are displayed in blue and cyan. All halves of cut animals continue locomotion after the cut without a noticeable pause. Note, halves of the same animal turn synchronously.

Supplementary Video S8 An example of the “hollow sphere” formation from the upper layer. The lower layer is responsible for locomotion and feeding.

Supplementary Video S9 An early stage of the “hollow sphere” separation from *Trichoplax adhaerens*.

Supplementary Video S10 An elongated form of *Trichoplax adhaerens* with two spherical dorsal structures.

Supplementary Video S11 An elongated form of *Trichoplax adhaerens* with four dorsal spheres.

Supplementary Video S12
*Trichoplax adhaerens* with two isolated hollow spheres.

Supplementary Video S13 Putative bacteria from damaged “sphere”.

Supplementary Video S14 Comparison of a swarmer and a free-floating “sphere”. The “solid” swarmer resembles a small juvenile animal with ciliated locomotion, and its interior is filled with cells, without a recognized cavity. A “hollow sphere” has a well-defined micro-cavity, no recognized behaviors, and a low density of ciliated cells on the surface (which might represent dorsal/upper epithelial cells; see also Thiemann and Ruthmann, 1988; 1990, 1991).
