# Supplementary figures and images for: Expanding of Life Strategies in Placozoa: Insights From Long-Term Culturing of Trichoplax and Hoilungia
Source: Front Cell Dev Biol. 2022 Feb 9;10:823283. doi: 10.3389/fcell.2022.823283 (PMC8864292; doi:10.3389/fcell.2022.823283)

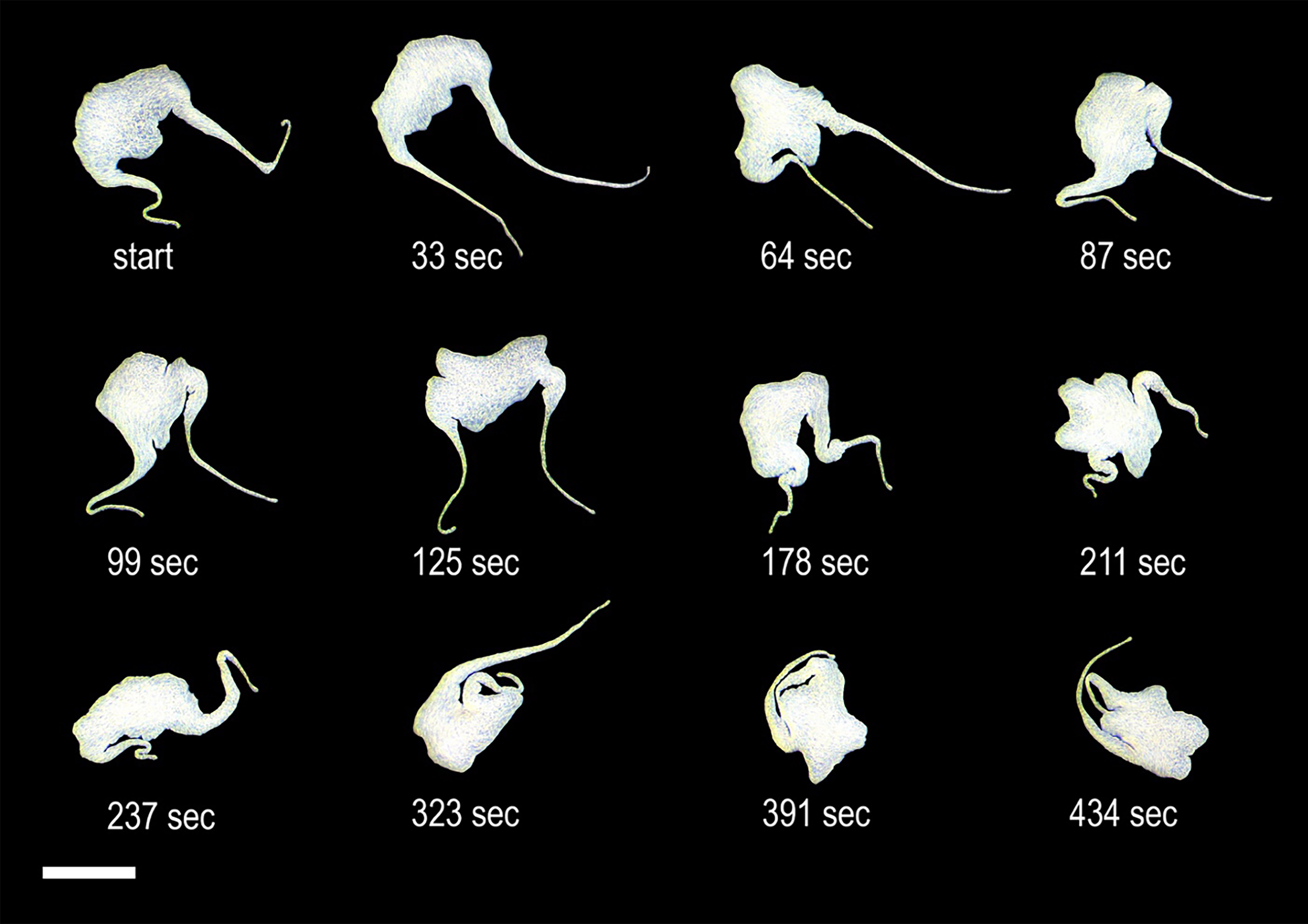

Supplement: Supplementary file 1 [file Image3.JPEG]

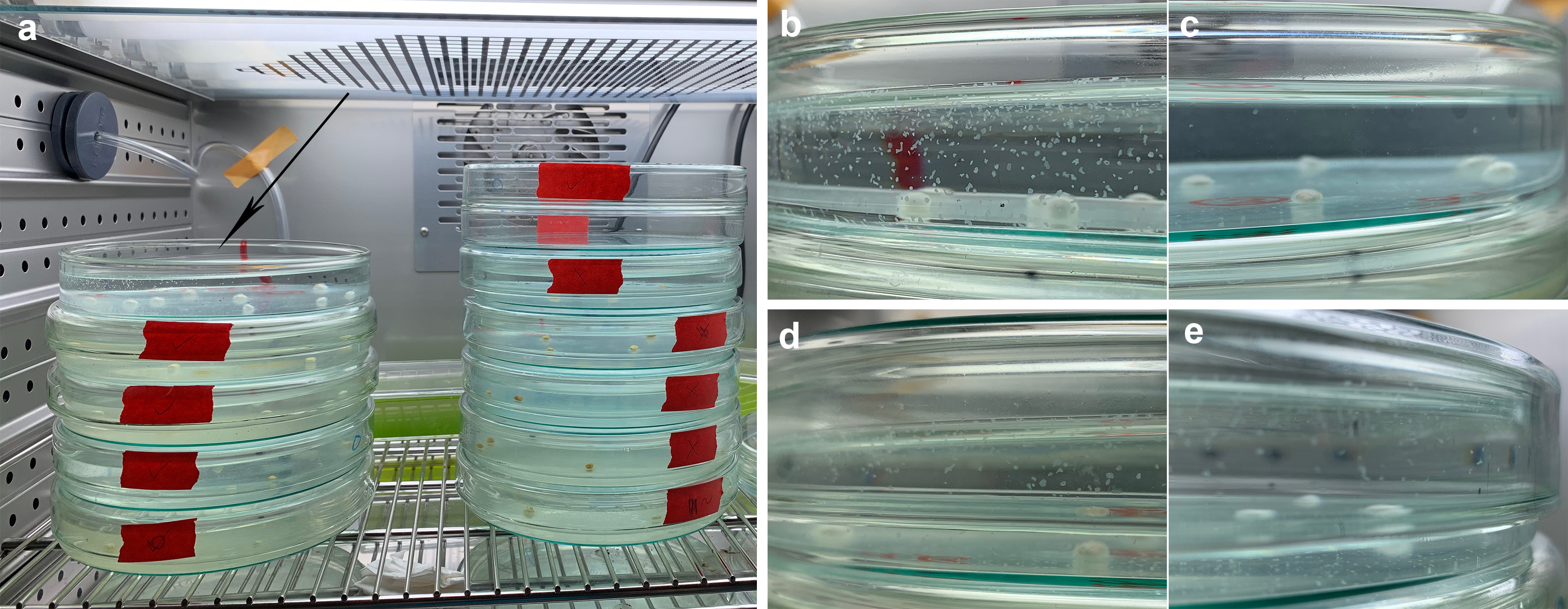

Supplement: Supplementary file 5 [file Image1.JPEG]

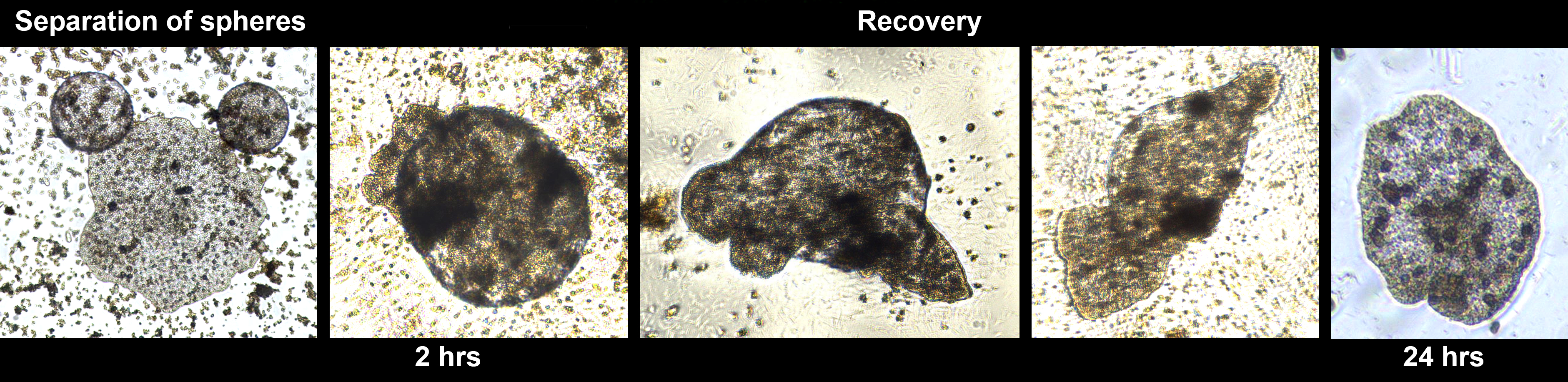

Supplement: Supplementary file 7 [file Image4.JPEG]

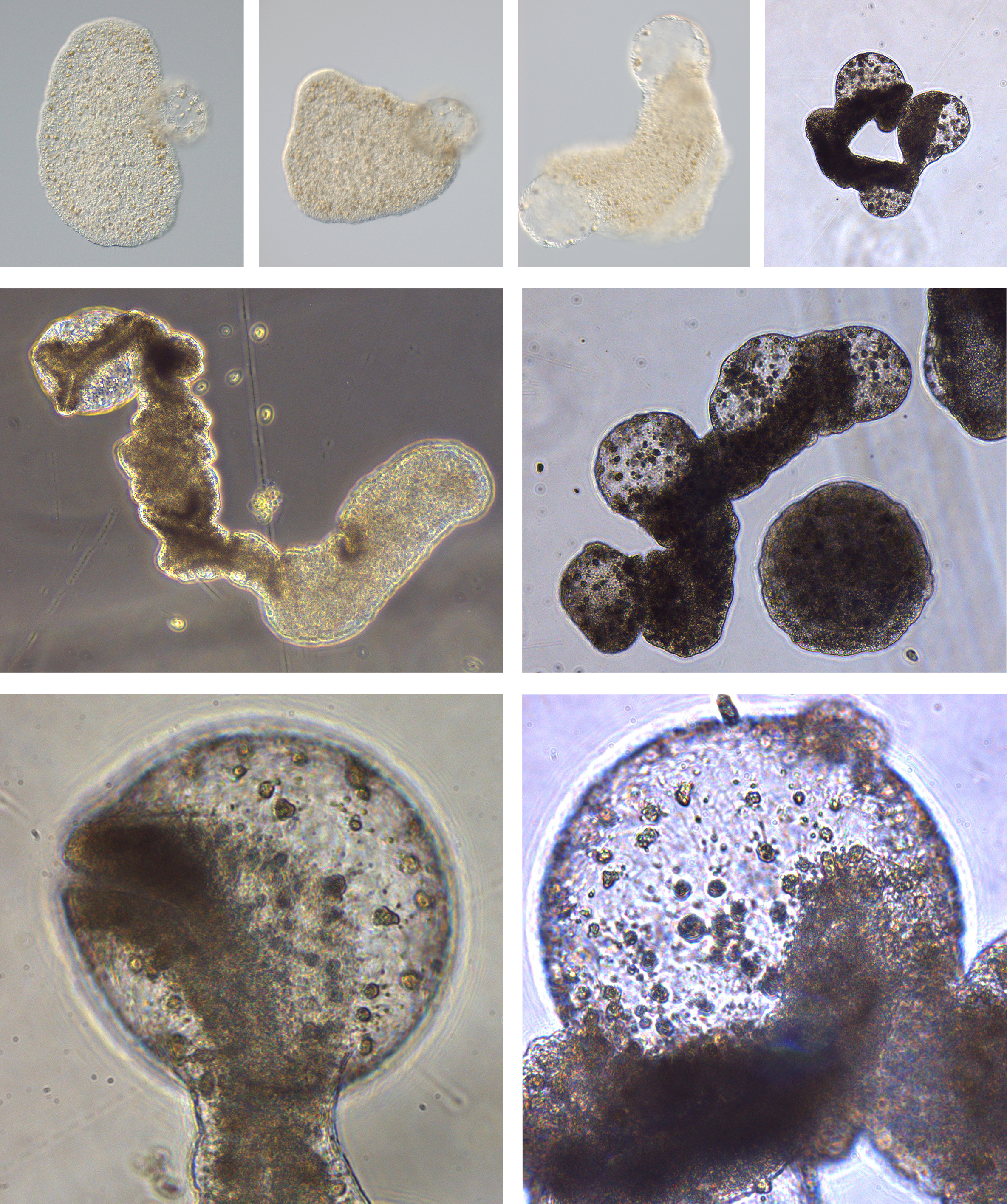

Supplement: Supplementary file 8 [file Image7.JPEG]

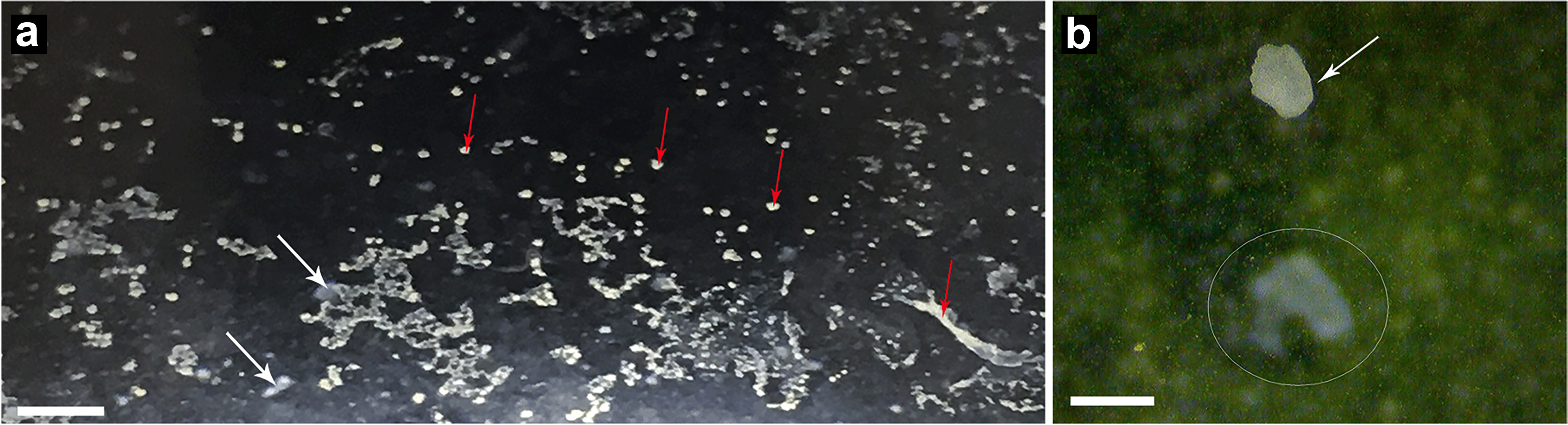

Supplement: Supplementary file 9 [file Image2.JPEG]

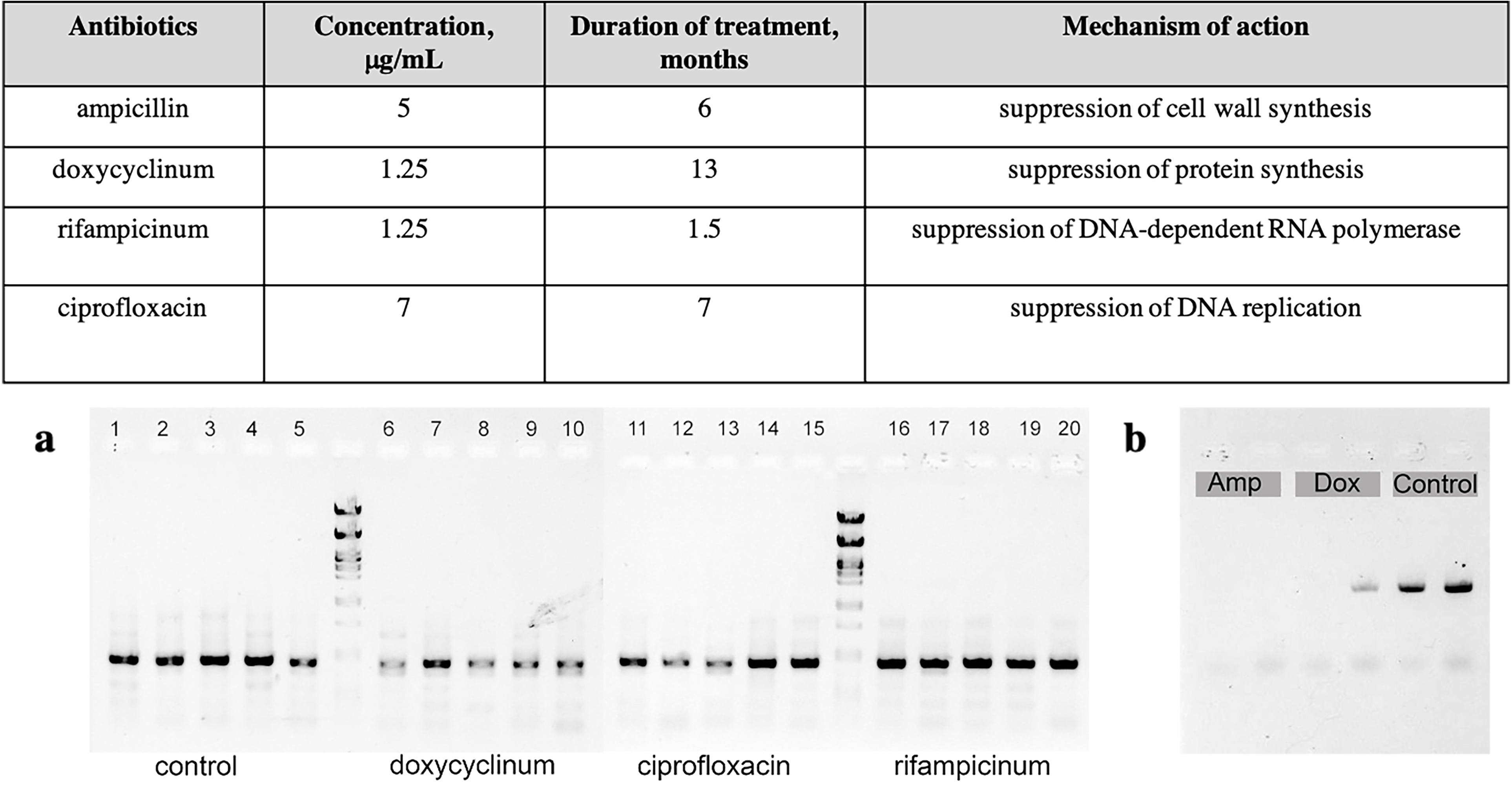

Supplement: Supplementary file 10 [file Image5.JPEG]

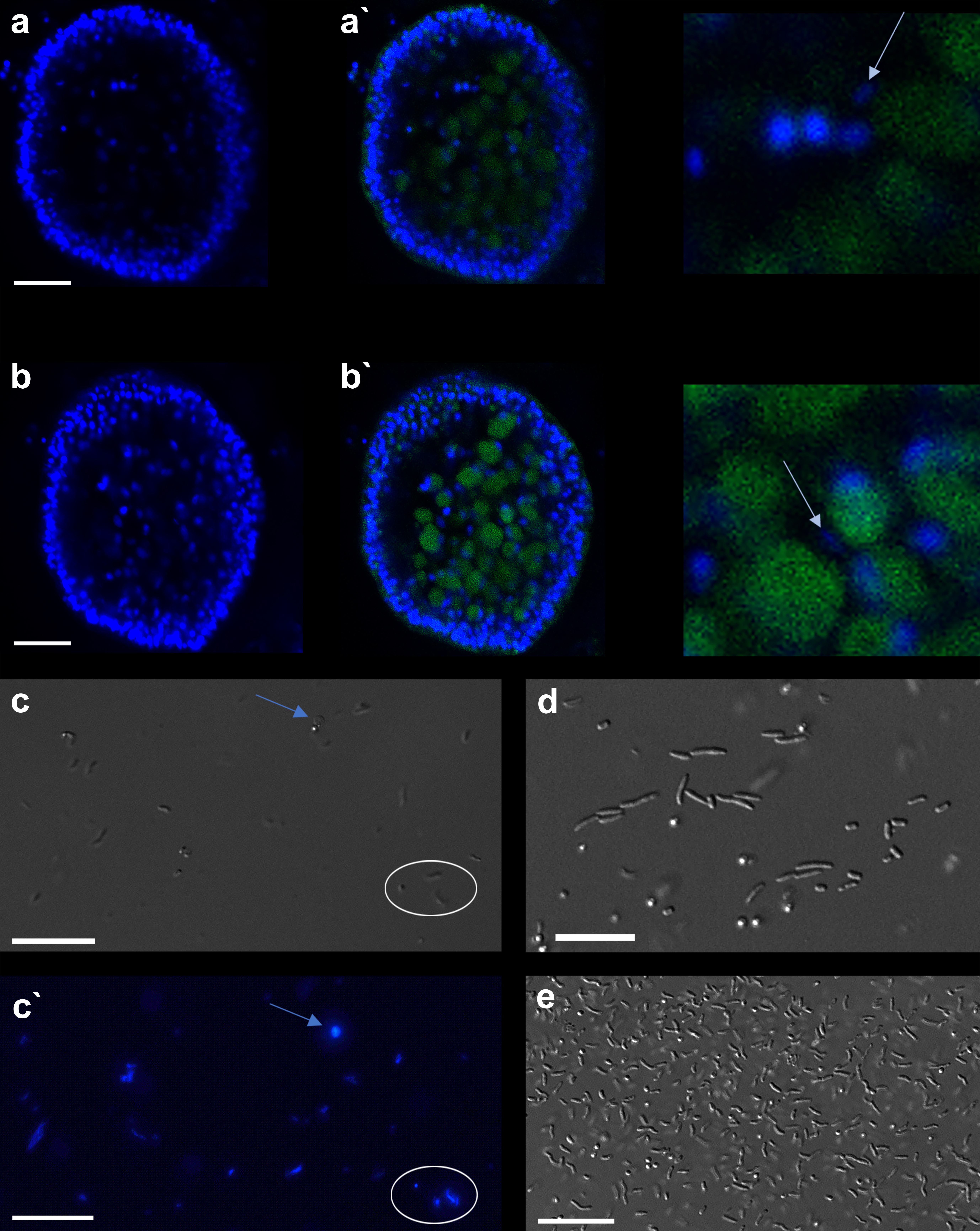

Supplement: Supplementary file 15 [file Image8.JPEG]

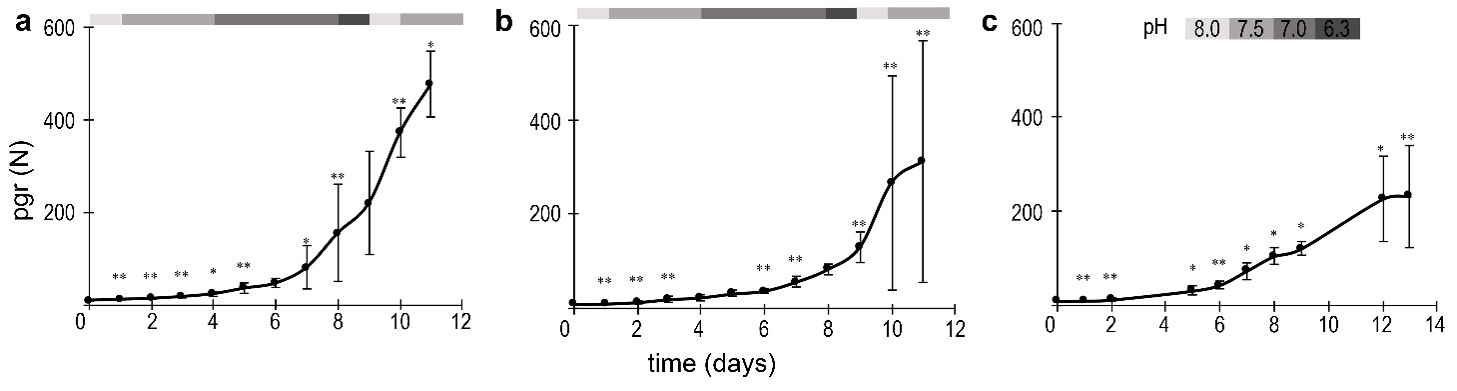

Supplement: Supplementary file 23 [file Image6.JPEG]
